# Advances in the Relationship Between Pyroptosis and Diabetic Neuropathy

**DOI:** 10.3389/fcell.2021.753660

**Published:** 2021-10-12

**Authors:** Jingyu Xu, Shufang Cai, Jiaxin Zhao, Ke Xu, Hao Ji, Chengbiao Wu, Jian Xiao, Yanqing Wu

**Affiliations:** ^1^Engineering Laboratory of Zhejiang Province for Pharmaceutical Development of Growth Factors, Biomedical Collaborative Innovation Center of Wenzhou, The Institute of Life Sciences, Wenzhou University, Wenzhou, China; ^2^School of Pharmaceutical Science, Wenzhou Medical University, Wenzhou, China; ^3^Clinical Research Center, Affiliated Xiangshan Hospital, Wenzhou Medical University, Wenzhou, China

**Keywords:** diabetes, neuropathy, pyroptosis, neuroinflammation, oxidative stress

## Abstract

Pyroptosis is a novel programmed cell death process that promotes the release of interleukin-1β (IL-1β) and interleukin-18 (IL-18) by activating inflammasomes and gasdermin D (GSDMD), leading to cell swelling and rupture. Pyroptosis is involved in the regulation of the occurrence and development of cardiovascular and cerebrovascular diseases, tumors, and nerve injury. Diabetes is a metabolic disorder characterized by long-term hyperglycemia, insulin resistance, and chronic inflammation. The people have paid more and more attention to the relationship between pyroptosis, diabetes, and its complications, especially its important regulatory significance in diabetic neurological diseases, such as diabetic encephalopathy (DE) and diabetic peripheral neuropathy (DPN). This article will give an in-depth overview of the relationship between pyroptosis, diabetes, and its related neuropathy, and discuss the regulatory pathway and significance of pyroptosis in diabetes-associated neuropathy.

## Introduction

Diabetes is a metabolic disorder characterized by hyperglycemia and insulin resistance. Under hyperglycemia condition, advanced glycosylation end products (AGEs) and reactive oxygen species (ROS) are significantly increased and promote a series of adverse reactions, such as chronic inflammation and oxidative stress, and eventually induce a series of complications. Pyroptosis is a newly discovered form of programmed cell necrosis. Different from the pathogenesis of apoptosis, pyroptosis is activated through the lysis of caspase-1/4/5/11 and activation of the gasdermin D (GSDMD) signaling pathway, as well as the release of inflammatory cytokines, interleukin-1β (IL-1β), and interleukin-18 (IL-18), which ultimately leads to the cell swelling and rupture. At present, a large number of studies showed that pyroptosis is an important regulatory mechanism of diabetic complications, especially in diabetic neuropathy (DN) (Zhan et al., 2020; Cheng et al., 2021). This article will systematically review the relationship between pyroptosis, diabetes, and DN, and provide the theoretical basis for further exploring the regulatory role of pyroptosis in DN.

## Pyroptosis

The discovery of pyroptosis will be traced back to 1986. Friedlander (1986) firstly found that treatment of anthrax lethal toxin (ALT) for primary macrophages of mouse can induce a large area of cell death and quickly release the contents. In 1992, the mice infected with the Gram-negative bacterium (*Shigella flexneri*) were found to have severe death of macrophage (Zychlinsky et al., 1992). The death of macrophages caused by these bacterial infection is dependent on caspase-1, but not caspase-3, that is required for apoptosis (Hilbi et al., 1998). In order to distinguish the cell apoptosis caused by DNA degradation, the condition of caspase-1 activation, release of pro-inflammatory factor, and cell swelling, rupture, and death after bacterial infection was named as “Pyroptosis” (Shi et al., 2017; Cookson and Brennan, 2001). The pyroptosis can be commonly divided into classical pathway and non-classical pathway.

### Classical Pathway

**FIGURE 1:**
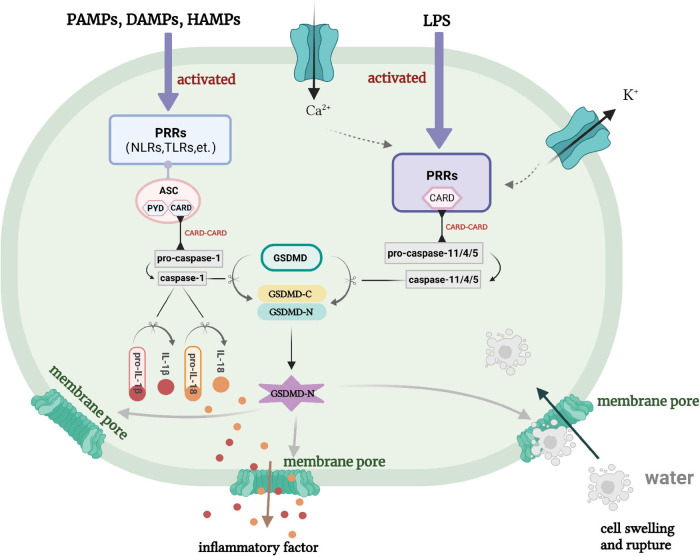
Classical pathway and non-classical pathway of pyroptosis. PRRs, pattern recognition receptors; ASC, apoptosis-associated speck-like protein containing a caspase-recruitment domain; LPS, lipopolysaccharide; NLRS, nucleotide-binding oligomerization domain-like receptors; PAMPS, pathogen-associated molecular patterns; DAMPS, danger-associated molecular patterns; HAMPS, homeostasis-altering molecular processes.

When the body is stimulated by danger signals, it will assemble caspase-1 and activate inflammasomes, then use GSDMD as a physiological substrate to promote the degradation of cell membranes (He et al., 2015), lastly lead to cell death and the release of mature IL-1β and IL-18 (Ali et al., 2017; Błażejewski et al., 2017). This caspase-1-dependent pyroptosis is known as the “classical pathway” (Figure 1). The assembly of inflammasomes is a key step in the process of pyroptosis. Inflammasome can recognize pathogen-associated molecular patterns (PAMPs), danger-associated molecular patterns (DAMPs), and homeostasis-altering molecular processes (HAMPs) through pattern recognition receptors (PRRs) (Liston and Masters, 2017; Silveira et al., 2017). After that, PRRs are activated and recruit the adaptor apoptosis-associated speck-like protein containing a caspase-recruitment domain (CARD) (ASC) (Yu et al., 2020). ASC contains two domains: pyrin domain (PYD) and CARD (Xue et al., 2019). Therefore, recruitment of ASC can cause homotype CARD–CARD interaction to each other, which promotes PRRs to combine with pro-caspase-1 and become a mature caspase-1. In addition, PRRs containing CARD can directly bind to CARD of pro-caspase-1 to form an ASC-free inflammasome complex (Hoss et al., 2017). Now, a variety of PRRs have been found to exist in the composition of inflammasomes, such as nucleotide-binding oligomerization domain-like receptors (NLRs) protein family, Toll-like receptors (TLRs) and retinoic acid inducible gene I-like receptors (RLRs), etc. (Jaeger et al., 2015). Activation of inflammasomes will promote the accumulation of GSDMD. Subsequently, active caspase-1 will cleave GSDMD to form C-gasdermin domain and N-gasdermin domain (He et al., 2015). At the same time, caspase-1 secures pro-IL-1β and pro-IL-18 to become the mature IL-1β and IL-18, then secretes into the cell and promotes inflammatory response (Itani et al., 2016). The active N-gasdermin domain in mammalian cells aggregates on the cell membrane and combines with lipids to form a large number of pores with a diameter of about 10–21 nm (Chen et al., 2016; Liu et al., 2016; Sborgi et al., 2016). The formation of these pores destroys the intracellular osmotic pressure, leads to cells swelling and eventually dissolution, resulting in cell pyroptosis. Due to the N-gasdermin domain of GSDMD has a pore-forming activity on cell membrane (Kovacs and Miao, 2017), pyroptosis is also defined as gasdermin-mediated programmed necrosis (He et al., 2015).

### Non-classical Pathway

Lipopolysaccharide (LPS) directly connects with caspase-11 to decompose GSDMD precursor form (pro-GSDMD) to form N-gasdermin, then induces the formation of membrane pore, which is named as “non-classical pathway” (Figure 1; Yokoyama et al., 2018). This may be because the intracytoplasmic PRRs are CARD-containing receptor proteins, which can recognize LPS, then recruit and aggregate the activated caspase-11 multi-protein complex (Kayagaki et al., 2011). In fact, caspase-11 itself can recognize LPS by binding to the specific and high-affinity lipid A of LPS (Knodler et al., 2014). This binding triggers the oligomerization of caspase-11, and activates its proteolytic activity. The caspase-4 and caspase-5 in human is homologous to caspase-11 in mice, which also directly binds to LPS (Casson et al., 2015; Viganò et al., 2015). Thus, LPS-induced activation of inflammasomes can also be happened in human innate immunity. There are studies showed that caspase-11 in mice or caspase-4/5 in human can combine with LPS and activate to cleave the GSDMD, result in the production of pores in the plasma membrane and K^+^ ion efflux, and ultimately triggering the activation of NLRP3 inflammasome (Baker et al., 2015; Rühl and Broz, 2015).

## The Regulatory Role of Diabetes on Pyroptosis During Neuropathy

Pyroptosis is a double-edged sword to the homeostasis of body. On the one hand, it helps to protect the multicellular organisms from bacterial infections; on the other hand, excessive pyroptosis may also lead to chronic inflammation (Lu et al., 2020). It is found that excessive pyroptosis leads to rapid rupture of plasma membrane, and excessive release of pro-inflammatory cytokines and chemokines, such as tumor necrosis factor-α (TNF-α), IL-1β, and interleukin-6 (IL-6), leading to neuroinflammation (Ramesh et al., 2013). Neuroinflammation triggers a series of secondary injuries after neuropathy and eventually leads to neuronal death (Simon et al., 2017). The levels of IL-1β and IL-18 were significantly elevated in cerebrospinal fluid, brain tissue, and plasma of patients with the infection of central nervous system (CNS), brain injury, and neurodegenerative diseases (Licastro et al., 2000; Huang et al., 2004). Then, the IL-1β and IL-18 can bind to their receptors on microglial cells, astrocytes, neurons, and endothelial cells, respectively, trigger a series of complex cascade reactions, and lead to the expression of inflammation-related gene. There have been more and more evidences suggesting that pyroptosis leads to neuronal death and aggravates the disease process in neurological diseases, such as ischemic stroke, cognitive impairment, spinal cord injury (SCI), and peripheral neuropathy, etc. (Liu et al., 2018). Pyroptosis is also an important regulatory mechanism in the occurrence and development of diabetes and its complications (Mamun et al., 2021). Diabetes is accompanied by chronic inflammation. Pyroptosis happens to be an inflammatory-mediated programmed cell death. Diabetes with long-term inflammation can cause excessive pyroptosis in the body (Wang et al., 2020). It had been reported that hyperglycemia can activate PYD containing NLRP3 inflammasome, and promote the transformation of pro-caspase-1 to caspase-1, thereby expedite the development of pyroptosis (Schroder et al., 2010). It was demonstrated that hyperglycemia initiates caspase-11/4 and GSDMD-dependent pyroptosis, and then leads to podocyte loss during diabetic nephropathy (Cheng et al., 2021).

**FIGURE 2:**
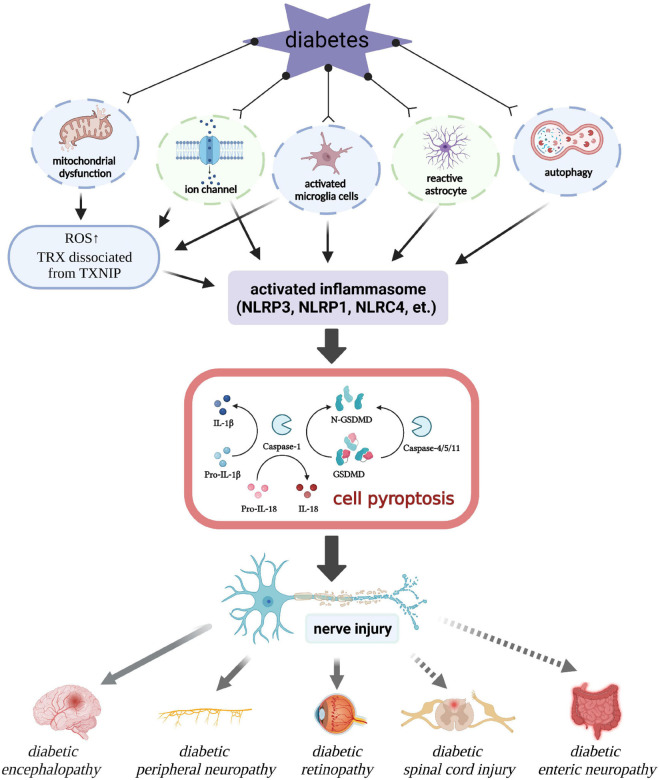
Regulatory role of pyroptosis in diabetic neuropathy. ROS, reactive oxygen species; TRX, thioredoxin; TXNIP, thioredoxin-interacting protein.

Pyroptosis may also be an important regulatory target of DN. Persistent inflammation caused by excessive secretion of pro-inflammatory factors and peroxides is an important pathological mechanism underlying DN. Therefore, pyroptosis is very likely to be a key factor during the occurrence and development of DN. Here, we will review the regulatory mechanism of pyroptosis in DN as following (Figure 2).

### Mitochondrial Dysfunction-Induced Oxidative Stress

Normal mitochondrial function plays a crucial role in maintaining the cellular homeostasis of body (Sas et al., 2018). Mitochondrial dysfunction produces a large amount of ROS. ROS is considered to be a key regulator of inflammatory response, which significantly promotes the activation of NLRP3 inflammasome in neurological diseases (Zhou et al., 2016). Gao et al. (2019) found that echinacoside (ECH) significantly reduces ROS level, improves the potential of mitochondrial membrane, blocks nuclear factor kappa-B (NF-κB) activation, then inhibits the activation of NLRP3 inflammasome, and lastly promotes locomotor functional recovery of rat after SCI. Additionally, diabetes-associated hyperglycemia, hyperinsulinemia, and insulin resistance results in mitochondrial damage, excessive production of ROS, and ultimately leads to redox imbalance in cells, which is the important pathological mechanism of diabetes-associated complications (Shi et al., 2020). It is reported that the co-treatment of high glucose and LPS can promote the production of ROS, aggravate NLRP3 inflammasome and caspase-1-dependent pyroptosis in H9C2 cardiomyocyte (Qiu et al., 2019). Moreover, excessive ROS may cause the dissociation of thioredoxin-interacting protein (TXNIP) and thioredoxin (TRX) (Strowig et al., 2012). Then, TXNIP combines with NLRP3 through a leucine-rich repeat domain (LRR) to form an active inflammasome complex (Han et al., 2018). The overexpression of TXNIP induces the activation of NLRP3 inflammasome in the injured spinal cord and triggers the neuronal cell death dependent on pyroptosis (Yanagisawa et al., 2019). It has been proposed that galectin-3 (Gal-3) can promote the neuroinflammatory response in SCI model by regulating the ROS/TXNIP/NLRP3 signaling pathway (Ren et al., 2019). It has also been found that Loganin inhibits the formation and activation of NLRP3 inflammasome and reduces pyroptosis by inhibiting oxidative stress and NF-κB–P2X_7_R–TNXIP signaling pathway in diabetic peripheral neuropathy (DPN) (Cheng et al., 2020). In our previous studies, we also found that mitochondrial damage-induced excessive ROS level is an important induction mechanism of diabetes-induced nerve damage, such as diabetes-associated cognitive dysfunction (Wang B. N. et al., 2021), diabetic embryonic neural tube malformation (Wang F. et al., 2017), and DPN (Li et al., 2021). Thus, the improvement of mitochondrial function and inhibition of oxidative stress are of great significance to the reduction of pyroptosis, the survival of nerve cells, and the recovery of nerve injury under diabetic condition.

### Microglial Activation

During the occurrence and development of neurological diseases, a variety of cells in the nervous system participate in the regulation of pyroptosis, such as macrophages/microglia and astrocytes. Microglial activation is found to initiate and maintain the inflammatory response during brain diseases, such as infectious nerve injury, acute CNS damage, and neurodegenerative diseases (Kumar et al., 2016; Xian et al., 2016; Song et al., 2017). Microglial cells are macrophage-like cells that reside in the CNS, which are the main cellular mediator of innate immune response (David et al., 2015) and pyroptosis (Vande Walle and Lamkanfi, 2016) after injury. They will continuously monitor the designated brain/spinal cord area, and participate in the regulation of the development of the CNS and later neuroprotective processes (Nayak et al., 2014). Microglial cells express a large amount of PRRs, which is responsible for the early identification of PAMPs and DAMPs, such as TLR, NLR, and triggering receptor expression on myeloid cells 2 (TREM2) (Fu et al., 2014). Over-activation of microglial cells promote the over-production of cytotoxic factors, such as TNF-α, IL-1β, IL-18, IL-6, ROS, and nitric oxide (NO), etc., then, it will be converted to M1 type and lead to neuroinflammation, oxidative stress and neuronal dysfunction (Nayak et al., 2014). Our previous study also found that microglial cells were activated after SCI, and their secreted proinflammatory factors (TNF-α and 1L-6) and M1 phenotypic marker protein (CD86) were significantly increased in the spinal cord from SCI group (Wu et al., 2021). Inhibition of microglial activation has a positive effect in the ameliorating pyroptosis and protecting neurons. MCC950, a selective inhibitor of NLRP3 inflammasome, significantly reduces neuroinflammation, brain edema, pyroptosis, and neurological dysfunction by inhibiting microglial activation in animals with brain trauma (Ismael et al., 2018; Xu K. Y. et al., 2018). In addition, CD73 [also known as ecto-5′-nucleotidase (NT5E)], an AMP hydrolase, is also found to inhibit the activation of NLRP3 inflammasome and GSDMD, reduce the pyroptosis of microglial cells, and play an important anti-neuroinflammatory effect in SCI recovery (Xu et al., 2021; Xu et al., 2021). The excessive activation of microglial cells is also a principal element of DN. Overaggregation of microglial cells is observed in both non-diseased and diseased regions of patients with diabetes mellitus complicated with acute cerebral infarction (Li et al., 2011). Moreover, hyperglycemia can activate NF-κB to increase the inflammatory response of microglial cells around the injury area of spinal cord, and aggravate secondary injury after SCI in diabetic mice (Kobayakawa et al., 2014). It is also found the significantly increase of activated microglial cells in the brain of APP/PS1xdb/db mouse (Ramos-Rodriguez et al., 2015). Moreover, high glucose stimulation can also activate NLRP3 inflammasome, trigger the pyroptosis of retinal microglial cell and lead to diabetic retinopathy (DR) (Huang et al., 2021). In a word, the over-activation of microglial cells is an important regulatory mechanism for pyroptosis in DN. Therefore, inhibition of microglial overactivation may be an important way to inhibit excessive pyroptosis in the process of DN.

### Astrocyte Activation

In addition to macrophages/microglia, astrocytes are also the important effector of inflammation in the nervous system (Karve et al., 2016; Shrivastava et al., 2017). Astrocytes have a series of functions, including glutamate uptake, synapse formation, structural support, and immune defense (Barreto et al., 2011). After injury, astrocytes show the features of hypertrophy, proliferation, and form the long protuberance, which will extend to the severely injured site and participate in the formation of dense astrocyte scar (Cregg et al., 2014). Maintaining the normal function of astrocytes plays an important role in the nerve function and repair after injury, especially in the diabetic environment. In a mouse model of type 2 diabetes, it is found that excessive activation of astrocytes in spinal cord can lead to mechanical allodynia (Liao et al., 2011). Reactive astrocytes can produce a large amounts of pro-inflammatory mediators to exacerbate the inflammatory response. The inflammasomes, such as NLRP1, NLRP3, NLRP6, and NLRC4, have been found in astrocytes (Walsh et al., 2014; Gustin et al., 2015; Freeman et al., 2017; Zhang et al., 2020). Recent studies further found that the difficulty in SCI recovery and thromboembolic stroke is closely related to the excessive activation of the NLRP1 inflammasome in astrocytes (Abulafia et al., 2009; de Rivero Vaccari et al., 2012). Moreover, Zhang et al. (2020) also found that the expression of NLRP6 in astrocytes is significantly increased under oxygen glucose deprivation/reoxygenation (OGD/R) condition, and leads to the excessive inflammation and pyroptosis of nerve cells. Chronic hyperglycemia is shown to activate astrocytes, interfere with the normal nerve growth, and induce the activation of inflammasomes (Rahman et al., 2020). This result suggests that activate astrocytes may be likely to be an important target during diabetes leading to the cell pyroptosis in nervous system. Thus, it is worth further exploring whether inhibition of astrocyte activation is a potential therapeutic strategy for DN treatment.

### Ion Channel

The imbalance of intracellular ion homeostasis is a common caused event of cell death, which has been found to have a certain relationship with pyroptosis. It has been reported that K^+^ efflux is involved in the activation of inflammasomes (Gong et al., 2018). Purinergic ligand-gated ion channel seven receptor (P2X_7_R) is a ligand-gated ion channel that mediated by potassium ion carriers and ATP (Alves et al., 2014). The P2R_7_X ion channel can activate the assembly of inflammasomes and subsequent caspase-1 through triggering K^+^ efflux and Ca^2+^ influx, and amplify the pro-inflammatory signals (Savio et al., 2018). Previous studies have shown that deletion of P2X_7_R reduces the incidence of diabetes, indicating that P2X_7_R may be an important target to reduce the occurrence and development of diabetes-related complications (Zhang et al., 2018). Overexpression of P2X_7_R can result in K^+^ efflux and increase membrane permeability in db/db mouse periodontitis model and LPS stimulated macrophages model, thereby increasing the possibility of pyroptosis (Karmakar et al., 2016; Zhou et al., 2020). In addition, P2X_7_R/NLRP1/caspase-1 is also one of the important pathway of inflammatory cascade in damaged neurons from diabetic patients. In the study of diabetic hippocampal neuron damage, it is proposed that the inhibition of P2X_7_R/NLRP1/caspase-1 pathway can alleviate nerve damage (Jing et al., 2021). Moreover, there is also evidence that the initiation of P2X_7_R ion channel is also a key regulatory factor in the microglial activation. Overexpression of P2X_7_R is found and involved in the microglial activation in primary cultured hippocampal microglial cells (Monif et al., 2010), which may be a another pathway to trigger pyroptosis. In summary, the P2X_7_R ion channel may also be an important target for regulating pyroptosis in DN.

The transient receptor potential melastatin-related 2 (TRPM2) channel is also involved in the abnormal activity of Ca^2+^ ion channel. TRPM channel, a non-selective cation channel, plays a vital role in the process of development, proliferation, and death of cell. It has been reported that high glucose can induce oxidative stress through TRPM2 channel to promote Ca^2+^ influx (Wuensch et al., 2010). Under high glucose condition, the TRPM2 channel mediated promotion of Ca^2+^ influx is closely related to the overproduction of ROS and TXNIP-mediated activation of NLRP3 inflammasome (Tseng et al., 2016). TRPM2 can also inhibit LPS-induced pyroptosis in macrophages by inhibiting the production of ROS (Wang H. et al., 2017). Recently, DN has been paid more and more attention. TRPM channel may also be a potential target for reducing the oxidative stress and subsequently the activation of NLRP3 inflammasome during diabetes associated nerve injury.

### Autophagy

Autophagy is also involved in the regulation of pyroptosis. Autophagy activation is found to inhibit the activation of NLRP3 inflammasome in both brain injury (He et al., 2017) and liver injury (Han et al., 2016). In a normal organism, autophagy can reduce the occurrence and development of pyroptosis through removing damaged mitochondria and inhibiting the maturation and secretion of IL-1β and IL-18 (Nakahira et al., 2011; Yang F. et al., 2019). There is a study showed that autophagy activation by adrenomedullin (ADM) can promote the ROS–AMPK–mTOR axis and consequently alleviate pyroptosis in interstitial cells (Li et al., 2019). In addition, the relationship between autophagy and pyroptosis has also been payed more and more attention in diabetes-associated complications. It has been reported that metformin can activate AMPK/autophagy to inhibit the activation of NLRP3 inflammasome and reduce pyroptosis, thereby exerting cardioprotective and anti-inflammatory effects in diabetic cardiomyopathy (Yang F. et al., 2019). At the same time, the study also indicates that mild hypothermia may inhibit the pyroptosis through enhancing autophagy after diabetic cerebral ischemia, thus alleviating brain injury (Tu et al., 2019). However, the molecular mechanism underlying diabetes-induced autophagy to reduce pyroptosis needs to be further investigated. Overall, autophagy may also be key regulatory factor between DN and pyroptosis.

Base on the above studies, it suggests that diabetes regulate pyroptosis through nerve cells, mitochondria, ion channels, and autophagy, and thus participate in the regulation of nerve injury repair. These regulatory mechanisms are a systemically regulated process from the cellular level to the molecular level. They have mutual cross-talking among them rather than exist independently. For example, both ion channel destruction and mitochondrial function damage can promote oxidative stress. Moreover, the destruction of ion channel can directly activate pyroptosis by promoting mitochondrial function damage, which is not conducive to nerve injury repair in diabetic condition. Additionally, the action of diabetes on microglial cells and astrocytes also has a common characteristic, and excessive inflammatory response is the ultimate caused factor for the development of pyroptosis. Therefore, the treatment of DN requires comprehensive consideration of various factors, and comprehensively inhibits the occurrence and development of pyroptosis from the cellular level to the molecular level.

## Pyroptosis in Diabetic Neuropathy

Despite the continuous improvement of global medical standards, diabetes still affects the life quality of people of the world. According to the International Diabetes Foundation, more than 700 million people will be diagnosed with diabetes by 2045 (Che et al., 2020). Diabetes is a chronic metabolic abnormal disease, and long-term hyperglycemia can lead to a series of complications through a combination of mechanisms, such as excessive inflammation, increased AGE, elevated cellular stress, and mitochondrial damage. Diabetes leads to a variety of neurological diseases, such as diabetic encephalopathy (DE), diabetic neurodegeneration, and DPN. Chronic hyperglycemia has been shown to affect cognition, signal transduction, neurotransmission, and synaptic plasticity, leading to nerve damage or neuropathy (Chen et al., 2018; Fang et al., 2018). Pyroptosis is involved in the pathogenesis of DN (Figure 2). Next, we will review the relationship between pyroptosis and series of DN.

### Diabetic Encephalopathy

In recent years, there are more and more attention has been paid to diabetes-induced brain injury. Diabetes aggravates the inflammatory response after brain injury with manifested as neurodegeneration, cerebral infarction, and progressive cognitive decline (Erukainure et al., 2019; Liu et al., 2020). Compared with patients with normal brain injury, patients with DE have more severer disease, slower recovery, and higher mortality (Sun et al., 2016). Some studies have shown that microglial cells play a specific role in neuronal injury after ischemic injury. Excessive activation of microglial cells produces a large amount of pro-inflammatory cytokines, lead to neuron necrosis and apoptosis, thereby exacerbating diabetic cerebral ischemia/reperfusion (I/R) injury (Huang et al., 2015). Furthermore, it is found that lncRNA-Fendrr is highly expressed in diabetic brain I/R model and microglial cells treated with high glucose or hypoxia/reoxygenation (H/R), which protects the ubiquitination and degradation of NLRC4 protein through E3 ubiquitin ligase HERC2, increases the pyroptosis of microglial cells, and thus aggravates diabetic brain I/R damage (Wang L. Q. et al., 2021). These studies indicate that pyroptosis plays a important role in the occurrence and development of DE, especially the NLRP3 inflammasome pathway. There are some studies showed that STZ-induced diabetic mice cause hippocampal neuronal apoptosis and pyroptosis in an NLRP3-dependent manner, and lead to depression-like behavior (Li et al., 2020). In the research on the therapeutic effect of melatonin, it was found that melatonin can regulate the miR-214-3p/caspase-1 and miR-214-3p/ATG12 pathways, and inhibit the neuronal pyroptosis and excessive autophagy with the evidence of decreased levels of NLRP3, caspase-1, GSDMD-N, IL-1β, LC3, Beclin 1, and ATG12 (Che et al., 2020). Hong et al. (2018) also found that MCC950, a specific inhibitor of NLRP3, can improve cerebral I/R injury in diabetic mice and increase the survival rate of diabetic ischemic stroke mice. Moreover, it was found that hyperglycemia promotes the assembly and activation of NLRP1 inflammasome, and initiates neuroinflammation and neuronal damage (Meng et al., 2014), suggesting NLRP1 may also be a potential treatment to prevent hyperglycemia-related brain damage, which is further confirmed by Jing et al. (2021) study. They found that Naofucong can significantly inhibit the P2 × 7R/NLRP1/caspase-1 pathway and improve the HT22 hippocampal neuron damage induced by high glucose (Jing et al., 2021). Furthermore, in the studying of the gestational diabetes, it was showed that the aggregation of chemotactic proteins in the offspring’s brain tissue induces the neuronal loss, increases the aggregation of macrophages, and activates the pyroptosis of macrophages in a ChemR23-dependent manner, which leads to neurological damage and cognitive impairment in the offspring (Liang et al., 2019). In a word, DE is an extremely complicated process, and the specific role of pyroptosis in various of encephalopathy needs to be further explored (Table 1). However, it is certain that NLRP3 and NLRP1 are important caused factors in the development of DE. Therefore, targeting the inflammasomes, especially NLRP3, may be an important strategy for DE treatment, and NLRP3 inhibitors, such as OLT1177 or CY-09, can be selected to further explore their function in DE.

**TABLE 1:** Researches on the pyroptosis in diabetic encephalopathy.

**Diseases**	**The mechanism that triggering pyroptosis**	**Inflammasomes**	**References**
Diabetic cerebral I/R model	Activated and induced microglial pyroptosis	NLRC4	Wang L. Q. et al., 2021
Cognitive disorder in offspring of diabetes	Aggregation of macrophages caused by the aggregation of chemotactic proteins	NLRP3	Liang et al., 2019
Diabetic cognitive impairment	P2 × 7R/NLRP1/caspase-1	NLRP1	Jing et al., 2021
Diabetic depression	ROS overproduction	NLRP3	Li et al., 2020
Diabetic brain damage	miR-214-3p/caspase-1/ATG12 axis	NLRP3	Che et al., 2020
Diabetic cerebral ischemia	Activation of autophagy	NLRP3	Tu et al., 2019
Diabetic ischemic stroke	Activation of NLRP3 inflammasome	NLRP3	Hong et al., 2018
Diabetic brain injury	Pannexin 1 mediated the activation of NLRP1 inflammasome	NLRP1	Meng et al., 2014

### Diabetic Peripheral Neuropathy

Diabetic peripheral neuropathy is one of the most common complications of type 2 diabetes. DPN usually affects the sensory and motor neuron of peripheral nerve, and lead to symptoms such as lower extremity pain and foot ulcers, which seriously affects the life quality of patients (Yang H. et al., 2019). This is mainly due to hyperglycemia causing inflammation and damage of peripheral nerve, slowing the speed of nerve conduction, and leading to excessive release of ROS. Previous studies reported that paclitaxel can induce mitochondrial damage and ROS over-production in peripheral nerves accompanied by the activation of NLRP3 inflammasome and the infiltration of macrophages, leading to nerve pain (Jia et al., 2017). In DPN, hyperglycemia-induced the over-production of ROS is a recognized pathogenesis for DPN (Mrakic-Sposta et al., 2018). Thus, it is most likely that over-production of ROS activates NLRP3 inflammasome and triggers the pyroptosis during the development of DPN. Cheng et al. found Loganin can reduce the pyroptosis of Schwann cells (SCs) by reducing intracellular ROS and inhibiting the activation of NF-κB and NLRP3 inflammasome during DPN development (Cheng et al., 2020). These studies indicate that pyroptosis is involved in the occurrence and development of DPN. Inhibition of ROS and NLRP3 inflammasome activity are the important theoretical therapy for DPN. However, it is still need to further explore the potential target of pyroptosis in DPN treatment. Our study found that fibroblast growth factor 1 (FGF1) expression is significantly inhibited after DPN, and exogenous FGF1 could alleviate DPN by alleviating oxidative stress in SCs (Li et al., 2021). Moreover, FGF21 and FGF10 also show significant therapeutic potential in peripheral nerve injury, both of which can reduce oxidative stress and promote neurological recovery. FGFs have also been found to exert anti-pyroptosis property, especially FGF21 (Zeng et al., 2020). Therefore, we speculate that FGFs may be an choice for inhibiting pyroptosis and alleviating DPN.

### Diabetic Retinopathy (Optical Nerve)

As the prevalence of type 2 diabetes continuously increase, DR has affected nearly 93 million people worldwide (Abcouwer and Gardner, 2014). Neurodegeneration is an important caused event for DR, and neurosensory retina is considered to play an important regulatory role in DR development. Although DR has always been considered as a diabetic microvascular disease, recent studies showed that the retinal neurodegeneration precedes the microvascular dysfunction during DR development, and leads to microvascular abnormalities (Abcouwer and Gardner, 2014). DR is generally divided into two stages: early non-proliferative DR and later proliferative DR (Lutty, 2013). In the early non-proliferative DR, it will occur neuroinflammation, capillary loss, glial cells activation, and increased vascular permeability, thereby impairing visual function and partly contributing to vascular abnormalities in the later proliferative DR (Lutty, 2013; Abcouwer and Gardner, 2014). A large number of basic and clinical researches indicate that retinal neurons and glial cells profoundly affect the retinal microvascular system (Moran et al., 2016). At present, neurovascular units is used to describe the mutual application of neurons, glial cells, and vascular-related cells that regulate retinal blood flow, structure, and function in the retina (Metea and Newman, 2007). Diabetes affects the entire neurovascular unit of the retina. High glucose-mediated the continuous activation of glial cells leads to the loss of retinal ganglion, microvascular dysfunction, and neurodegeneration (Cuenca et al., 2014). Huang et al. (2021) found that high glucose induces the pyroptosis of retinal microglial cells by activating NLRP3 inflammasome. Another study also showed that *Nlrp3* gene knockout down-regulates the expression of caspase-1 and pro-inflammatory cytokines, and reduces the death of retinal ganglion cells after crush injury of optic nerve (Puyang et al., 2016). Moreover, high glucose significantly induces elevated levels of caspase-1 and IL-1β in Müller cells and microglial cells during DR (Feenstra et al., 2013). A large number of studies report the phenomenon of pyroptosis in endothelial cells (Gu et al., 2019) and pericytes (Gan et al., 2020; Yu et al., 2021) during DR development. The pyroptosis of microglial cells or neuron in early stage will create a inflammatory microenvironment of the neurovascular unit, which may be the main inducer for the pyroptosis in endothelial cells and pericytes. These studies suggest that pyroptosis plays an important role in the development of DR, and early prevention of pyroptosis during neuropathy in the early stage of DR may be an important approach to treat late-stage microvascular abnormalities.

### Diabetes With Spinal Cord Injury

Spinal cord injury affects millions of people worldwide, which causes severe motor deficits in the area below the injured segment (Singh et al., 2014). After mechanical trauma, the spinal cord suffers local blood edema and neuroinflammation, followed by a series of secondary injuries, which enlarges the inflammatory response around the original injury center (Beattie, 2004). Increasing evidences suggest that the NF-κB pathway is triggered after SCI, and activates NLRP3 inflammasome (Zhou et al., 2016). It was observed that SCI enhances the expressions of pyroptosis-related protein markers (GSDMD, cleaved caspase-1 and cleaved caspase-11) and inflammatory mediators (IL-1β and IL-18) after SCI (Zendedel et al., 2016; Al Mamun et al., 2021). This indicates that pyroptosis plays a key role in SCI (Zheng et al., 2019). Due to the high incidence of diabetes, more and more studies have focused on the impact of diabetes on SCI repair (Lien et al., 2016). Diabetes with SCI is characterized by poor recovery ability and high mortality. The study in experimental animals and clinical data found that hyperglycemia can promote the progressive damage of neuronal function after SCI (Lavela et al., 2006). Although there are few reports on the pyroptosis in diabetes complicated with SCI, hyperglycemia is reported to increase the inflammatory response of microglial cells by activating NF-κB, aggravate the secondary injury, and thus suppress the recovery of SCI (Kobayakawa et al., 2014). NF-κB is reported to initiate the activation of NLRP3 inflammasome by inducing the expression of pro-IL-1β and NLRP3 (Zhong et al., 2016). In conclusion, it is well worth further studying the regulatory role of pyroptosis in diabetes mellitus with SCI.

### Enteric Neuropathy

Gastrointestinal motility is regulated by the internal and external nerves of the intestine (Al-Shboul, 2013). It has found that HFD and obesity are related to gastrointestinal motility disorders (Al-Shboul, 2013). Previous study also showed that diabetes or HFD can increase the dysfunction of intermuscular neurons mediated by elevated oxidative stress, leading to dyskinesia (Voukali et al., 2011). Moreover, the level of cleaved caspase-1 in the myenteric ganglion of obese subjects is higher than that in normal-weight subjects (Ye et al., 2020). In a study with western diet (WD) (a high-fat diet) mice as a model, caspase-11-dependent pyroptosis results in the loss of intestinal nerve cells, leading to the delayed colonic transport and reduced colonic diastolic response (Ye et al., 2020). These studies suggest that pyroptosis may be an important therapeutic target for diabetes-induced enteric neuropathy.

## Conclusion

Pyroptosis is a new mode of programmed cell death, its role in the regulation of DN has been pay more and more attention. The current studies have found that hyperglycemia-mediated chronic inflammation, oxidative stress, mitochondrial damage, ROS overproduction, and ion channel dysfunction may be the molecular mechanism that initiates the pyroptosis, nerve injury, and lastly neurological dysfunction. Undoubtedly, blood glucose control is the most critical and fundamental step in the treatment of DN. In addition to blood glucose control, some agents with anti-inflammatory and antioxidant properties, such as cytokine inhibitor, ROS inhibitor, and NLRP3 inflammasome inhibitor, can also be selected to treat DN. However, it is still unclear the specific molecular target that triggers pyroptosis and the regulatory mechanism of various cell pyroptosis in nerve system during diabetes neuropathy, which is the key problem that hinders the targeted therapy of DN. Thus, it needs to be further explore the interrelationship between pyroptosis, diabetes, and its related neuropathy, and provide a theoretical basis for the treatment of DN.

## Author Contributions

JYX wrote the manuscript. SC and JZ participated in literature collection and producing the figures. KX and HJ participated in literature collection and edited the manuscript. CW edited the manuscript. JX and YW conceived the study and wrote the manuscript. All authors reviewed and approved the final manuscript.

## Conflict of Interest

The authors declare that the research was conducted in the absence of any commercial or financial relationships that could be construed as a potential conflict of interest.

## Publisher’s Note

All claims expressed in this article are solely those of the authors and do not necessarily represent those of their affiliated organizations, or those of the publisher, the editors and the reviewers. Any product that may be evaluated in this article, or claim that may be made by its manufacturer, is not guaranteed or endorsed by the publisher.

## Abbreviations

DN: diabetic neuropathy
GSDMD: gasdermin D
AGEs: advanced glycosylation end products
ROS: reactive oxygen species
ALT: anthrax lethal toxin
PAMPs: pathogen-associated molecular patterns
DAMPs: danger-associated molecular patterns
HAMPs: homeostasis-altering molecular processes
PRRs: pattern recognition receptors
CARD: caspase-recruitment domain
ASC: apoptosis-associated speck-like protein containing a CARD
PYD: pyrin domain
NLRs: nucleotide-binding oligomerization domain-like receptors
TLRs: Toll-like receptors
RLRs: retinoic acid inducible gene I-like receptors
LPS: lipopolysaccharide
JNK: c-JunN-terminal kinase
ECH: echinacoside
TXNIP: thioredoxin-interacting protein
TRX: thioredoxin
LRR: leucine-rich repeat domain
Gal-3: galectin-3
TREM2: triggering receptor expression on myeloid cells 2
TNF- α: tumor necrosis factor- α
IL-1 β: interleukin-1 β
IL-18: interleukin-18
IL-6: interleukin-6
NO: nitric oxide
NT5E: ecto-5′-nucleotidase
SCs: Schwann cells
OGD/R: oxygen glucose deprivation/reoxygenation
P2 × 7R: purinergic ligand-gated ion channel seven receptor
ADM: adrenomedullin
TRPM2: transient receptor potential melastatin-related 2
I/R: ischemia/reperfusion
H/R: hypoxia/reoxygenation
DPN: diabetic peripheral neuropathy
WD: western diet
NF- κ B: nuclear factor kappa-B.

## References

[B1] Abcouwer S. F. Gardner T. W. (2014). Diabetic retinopathy: loss of neuroretinal adaptation to the diabetic metabolic environment. *Ann. N. Y. Acad. Sci.* 1311 174–190. 10.1111/nyas.12412 24673341 PMC4154702

[B2] Abulafia D. P. De Rivero Vaccari J. P. Lozano J. D. Lotocki G. Keane R. W. Dietrich W. D. (2009). Inhibition of the inflammasome complex reduces the inflammatory response after thromboembolic stroke in mice. *J. Cereb. Blood Flow Metab.* 29 534–544. 10.1038/jcbfm.2008.143 19066616

[B3] Al-Shboul O. A. (2013). The importance of interstitial cells of cajal in the gastrointestinal tract. *Saudi J. Gastroenterol.* 19 3–15. 10.4103/1319-3767.105909 23319032 PMC3603487

[B4] Al Mamun A. Wu Y. Monalisa I. Jia C. Zhou K. Munir F. (2021). Role of pyroptosis in spinal cord injury and its therapeutic implications. *J. Adv. Res.* 28 97–109. 10.1016/j.jare.2020.08.004 33364048 PMC7753222

[B5] Ali M. F. Dasari H. Van Keulen V. P. Carmona E. M. (2017). Canonical stimulation of the NLRP3 inflammasome by fungal antigens links innate and adaptive B-lymphocyte responses by modulating IL-1β and IgM production. *Front. Immunol.* 8:1504. 10.3389/fimmu.2017.01504 29170665 PMC5684107

[B6] Alves L. A. De Melo Reis R. A. De Souza C. A. De Freitas M. S. Teixeira P. C. Neto Moreira Ferreira D. (2014). The P2X7 receptor: shifting from a low- to a high-conductance channel - an enigmatic phenomenon? *Biochim. Biophys. Acta* 1838 2578–2587. 10.1016/j.bbamem.2014.05.015 24857862

[B7] Baker P. J. Boucher D. Bierschenk D. Tebartz C. Whitney P. G. D’silva D. B. (2015). NLRP3 inflammasome activation downstream of cytoplasmic LPS recognition by both caspase-4 and caspase-5. *Eur. J. Immunol.* 45 2918–2926. 10.1002/eji.201545655 26173988

[B8] Barreto G. E. Gonzalez J. Torres Y. Morales L. (2011). Astrocytic-neuronal crosstalk: implications for neuroprotection from brain injury. *Neurosci. Res.* 71 107–113. 10.1016/j.neures.2011.06.004 21693140

[B9] Beattie M. S. (2004). Inflammation and apoptosis: linked therapeutic targets in spinal cord injury. *Trends Mol. Med.* 10 580–583. 10.1016/j.molmed.2004.10.006 15567326

[B10] Błażejewski A. J. Thiemann S. Schenk A. Pils M. C. Gálvez E. J. C. Roy U. (2017). Microbiota normalization reveals that canonical caspase-1 activation exacerbates chemically induced intestinal inflammation. *Cell Rep.* 19 2319–2330. 10.1016/j.celrep.2017.05.058 28614717

[B11] Casson C. N. Yu J. Reyes V. M. Taschuk F. O. Yadav A. Copenhaver A. M. (2015). Human caspase-4 mediates noncanonical inflammasome activation against gram-negative bacterial pathogens. *Proc. Natl. Acad. Sci. U. S. A.* 112 6688–6693. 10.1073/pnas.1421699112 25964352 PMC4450384

[B12] Che H. Li H. Li Y. Wang Y. Q. Yang Z. Y. Wang R. L. (2020). Melatonin exerts neuroprotective effects by inhibiting neuronal pyroptosis and autophagy in STZ-induced diabetic mice. *FASEB J.* 34 14042–14054. 10.1096/fj.202001328r 32910484

[B13] Chen R. Shi J. Yin Q. Li X. Sheng Y. Han J. (2018). Morphological and pathological characteristics of brain in diabetic encephalopathy. *J. Alzheimers Dis.* 65 15–28. 10.3233/jad-180314 30040723

[B14] Chen X. He W. T. Hu L. Li J. Fang Y. Wang X. (2016). Pyroptosis is driven by non-selective gasdermin-D pore and its morphology is different from MLKL channel-mediated necroptosis. *Cell Res.* 26 1007–1020. 10.1038/cr.2016.100 27573174 PMC5034106

[B15] Cheng Q. Pan J. Zhou Z. L. Yin F. Xie H. Y. Chen P. P. (2021). Caspase-11/4 and gasdermin D-mediated pyroptosis contributes to podocyte injury in mouse diabetic nephropathy. *Acta Pharmacol. Sin.* 42 954–963. 10.1038/s41401-020-00525-z 32968210 PMC8149386

[B16] Cheng Y. C. Chu L. W. Chen J. Y. Hsieh S. L. Chang Y. C. Dai Z. K. (2020). Loganin attenuates high glucose-induced schwann cells pyroptosis by inhibiting ROS generation and NLRP3 inflammasome activation. *Cells* 9:1948. 10.3390/cells9091948 32842536 PMC7564733

[B17] Cookson B. T. Brennan M. A. (2001). Pro-inflammatory programmed cell death. *Trends Microbiol.* 9 113–114. 10.1016/s0966-842x(00)01936-3 11303500

[B18] Cregg J. M. Depaul M. A. Filous A. R. Lang B. T. Tran A. Silver J. (2014). Functional regeneration beyond the glial scar. *Exp. Neurol.* 253 197–207. 10.1016/j.expneurol.2013.12.024 24424280 PMC3951813

[B19] Cuenca N. Fernández-Sánchez L. Campello L. Maneu V. De La Villa P. Lax P. (2014). Cellular responses following retinal injuries and therapeutic approaches for neurodegenerative diseases. *Prog. Retin Eye Res.* 43 17–75. 10.1016/j.preteyeres.2014.07.001 25038518

[B20] David S. Greenhalgh A. D. Kroner A. (2015). Macrophage and microglial plasticity in the injured spinal cord. *Neuroscience* 307 311–318. 10.1016/j.neuroscience.2015.08.064 26342747

[B21] de Rivero Vaccari J. P. Bastien D. Yurcisin G. Pineau I. Dietrich W. D. De Koninck Y. (2012). P2X4 receptors influence inflammasome activation after spinal cord injury. *J. Neurosci.* 32 3058–3066. 22378878 10.1523/JNEUROSCI.4930-11.2012 PMC6622016

[B22] Erukainure O. L. Ijomone O. M. Sanni O. Aschner M. Islam M. S. (2019). Type 2 diabetes induced oxidative brain injury involves altered cerebellar neuronal integrity and elemental distribution, and exacerbated Nrf2 expression: therapeutic potential of raffia palm (*Raphia hookeri*) wine. *Metab. Brain Dis.* 34 1385–1399. 10.1007/s11011-019-00444-x 31201727

[B23] Fang F. Zhan Y. F. Zhuo Y. Y. Yin D. Z. Li K. A. Wang Y. F. (2018). Brain atrophy in middle-aged subjects with Type 2 diabetes mellitus, with and without microvascular complications. *J. Diabetes* 10 625–632. 10.1111/1753-0407.12646 29380932

[B24] Feenstra D. J. Yego E. C. Mohr S. (2013). Modes of retinal cell death in diabetic retinopathy. *J. Clin. Exp. Ophthalmol.* 4:298. 24672740 10.4172/2155-9570.1000298 PMC3963519

[B25] Freeman L. Guo H. David C. N. Brickey W. J. Jha S. Ting J. P. (2017). NLR members NLRC4 and NLRP3 mediate sterile inflammasome activation in microglia and astrocytes. *J. Exp. Med.* 214 1351–1370. 10.1084/jem.20150237 28404595 PMC5413320

[B26] Friedlander A. M. (1986). Macrophages are sensitive to anthrax lethal toxin through an acid-dependent process. *J. Biol. Chem.* 261 7123–7126. 10.1016/s0021-9258(17)38364-3 3711080

[B27] Fu R. Shen Q. Xu P. Luo J. J. Tang Y. (2014). Phagocytosis of microglia in the central nervous system diseases. *Mol. Neurobiol.* 49 1422–1434. 10.1007/s12035-013-8620-6 24395130 PMC4012154

[B28] Gan J. Huang M. Lan G. Liu L. Xu F. (2020). High glucose induces the loss of retinal pericytes partly via NLRP3-Caspase-1-GSDMD-mediated pyroptosis. *Biomed. Res. Int.* 2020:4510628. 32420343 10.1155/2020/4510628 PMC7201508

[B29] Gao S. Xu T. Guo H. Deng Q. Xun C. Liang W. (2019). Ameliorative effects of echinacoside against spinal cord injury via inhibiting NLRP3 inflammasome signaling pathway. *Life Sci.* 237:116978. 10.1016/j.lfs.2019.116978 31644893

[B30] Gong T. Yang Y. Jin T. Jiang W. Zhou R. (2018). Orchestration of NLRP3 inflammasome activation by ion fluxes. *Trends Immunol.* 39 393–406. 10.1016/j.it.2018.01.009 29452983

[B31] Gu C. Draga D. Zhou C. Su T. Zou C. Gu Q. (2019). miR-590-3p inhibits pyroptosis in diabetic retinopathy by targeting NLRP1 and inactivating the NOX4 signaling pathway. *Invest. Ophthalmol. Vis. Sci.* 60 4215–4223. 10.1167/iovs.19-27825 31618425

[B32] Gustin A. Kirchmeyer M. Koncina E. Felten P. Losciuto S. Heurtaux T. (2015). NLRP3 inflammasome is expressed and functional in mouse brain microglia but not in astrocytes. *PLoS One* 10:e0130624. 10.1371/journal.pone.0130624 26091541 PMC4474809

[B33] Han J. Bae J. Choi C. Y. Choi S. P. Kang H. S. Jo E. K. (2016). Autophagy induced by AXL receptor tyrosine kinase alleviates acute liver injury via inhibition of NLRP3 inflammasome activation in mice. *Autophagy* 12 2326–2343. 10.1080/15548627.2016.1235124 27780404 PMC5173275

[B34] Han Y. Xu X. Tang C. Gao P. Chen X. Xiong X. (2018). Reactive oxygen species promote tubular injury in diabetic nephropathy: the role of the mitochondrial ros-txnip-nlrp3 biological axis. *Redox Biol.* 16 32–46. 10.1016/j.redox.2018.02.013 29475133 PMC5842313

[B35] He Q. Li Z. Wang Y. Hou Y. Li L. Zhao J. (2017). Resveratrol alleviates cerebral ischemia/reperfusion injury in rats by inhibiting NLRP3 inflammasome activation through Sirt1-dependent autophagy induction. *Int. Immunopharmacol.* 50 208–215. 10.1016/j.intimp.2017.06.029 28683365

[B36] He W. T. Wan H. Hu L. Chen P. Wang X. Huang Z. (2015). Gasdermin D is an executor of pyroptosis and required for interleukin-1β secretion. *Cell Res.* 25 1285–1298. 10.1038/cr.2015.139 26611636 PMC4670995

[B37] Hilbi H. Moss J. E. Hersh D. Chen Y. Arondel J. Banerjee S. (1998). *Shigella*-induced apoptosis is dependent on caspase-1 which binds to IpaB. *J. Biol. Chem.* 273 32895–32900. 10.1074/jbc.273.49.32895 9830039

[B38] Hong P. Li F. X. Gu R. N. Fang Y. Y. Lai L. Y. Wang Y. W. (2018). Inhibition of NLRP3 inflammasome ameliorates cerebral ischemia-reperfusion injury in diabetic mice. *Neural Plast.* 2018:9163521. 29853850 10.1155/2018/9163521 PMC5941718

[B39] Hoss F. Rodriguez-Alcazar J. F. Latz E. (2017). Assembly and regulation of ASC specks. *Cell Mol. Life Sci.* 74 1211–1229. 10.1007/s00018-016-2396-6 27761594 PMC11107573

[B40] Huang L. Li G. Feng X. Wang L. (2015). 15d-PGJ2 reduced microglia activation and alleviated neurological deficit of ischemic reperfusion in diabetic rat model. *Biomed. Res. Int.* 2015:864509. 26844229 10.1155/2015/864509 PMC4710931

[B41] Huang L. You J. Yao Y. Xie M. (2021). High glucose induces pyroptosis of retinal microglia through NLPR3 inflammasome signaling. *Arq. Bras. Oftalmol.* 84 67–73. 33470344 10.5935/0004-2749.20210010 PMC12289150

[B42] Huang W. X. Huang P. Hillert J. (2004). Increased expression of caspase-1 and interleukin-18 in peripheral blood mononuclear cells in patients with multiple sclerosis. *Mult. Scler.* 10 482–487. 10.1191/1352458504ms1071oa 15471361

[B43] Ismael S. Nasoohi S. Ishrat T. (2018). MCC950, the selective inhibitor of nucleotide oligomerization domain-like receptor protein-3 inflammasome, protects mice against traumatic brain injury. *J. Neurotrauma* 35 1294–1303. 10.1089/neu.2017.5344 29295651 PMC5962912

[B44] Itani S. Watanabe T. Nadatani Y. Sugimura N. Shimada S. Takeda S. (2016). NLRP3 inflammasome has a protective effect against oxazolone-induced colitis: a possible role in ulcerative colitis. *Sci. Rep.* 6:39075. 27966619 10.1038/srep39075 PMC5155456

[B45] Jaeger M. Stappers M. H. Joosten L. A. Gyssens I. C. Netea M. G. (2015). Genetic variation in pattern recognition receptors: functional consequences and susceptibility to infectious disease. *Future Microbiol.* 10 989–1008. 10.2217/fmb.15.37 26059622

[B46] Jia M. Wu C. Gao F. Xiang H. Sun N. Peng P. (2017). Activation of NLRP3 inflammasome in peripheral nerve contributes to paclitaxel-induced neuropathic pain. *Mol. Pain* 13:1744806917719804. 28714351 10.1177/1744806917719804 PMC5562344

[B47] Jing G. Wang H. Nan F. Liu Y. Zhang M. (2021). Naofucong ameliorates high glucose induced hippocampal neuron injury through suppressing P2X7/NLRP1/caspase-1 pathway. *Front. Pharmacol.* 12:647116. 10.3389/fphar.2021.647116 34093185 PMC8173084

[B48] Karmakar M. Katsnelson M. A. Dubyak G. R. Pearlman E. (2016). Neutrophil P2X7 receptors mediate NLRP3 inflammasome-dependent IL-1β secretion in response to ATP. *Nat. Commun.* 7:10555. 26877061 10.1038/ncomms10555 PMC4756306

[B49] Karve I. P. Taylor J. M. Crack P. J. (2016). The contribution of astrocytes and microglia to traumatic brain injury. *Br. J. Pharmacol.* 173 692–702. 10.1111/bph.13125 25752446 PMC4742296

[B50] Kayagaki N. Warming S. Lamkanfi M. Vande Walle L. Louie S. Dong J. (2011). Non-canonical inflammasome activation targets caspase-11. *Nature* 479 117–121. 22002608 10.1038/nature10558

[B51] Knodler L. A. Crowley S. M. Sham H. P. Yang H. Wrande M. Ma C. (2014). Noncanonical inflammasome activation of caspase-4/caspase-11 mediates epithelial defenses against enteric bacterial pathogens. *Cell Host Microbe* 16 249–256. 25121752 10.1016/j.chom.2014.07.002 PMC4157630

[B52] Kobayakawa K. Kumamaru H. Saiwai H. Kubota K. Ohkawa Y. Kishimoto J. (2014). Acute hyperglycemia impairs functional improvement after spinal cord injury in mice and humans. *Sci. Transl. Med.* 6:256ra137. 10.1126/scitranslmed.3009430 25273098

[B53] Kovacs S. B. Miao E. A. (2017). Gasdermins: effectors of pyroptosis. *Trends Cell Biol.* 27 673–684. 10.1016/j.tcb.2017.05.005 28619472 PMC5565696

[B54] Kumar A. Barrett J. P. Alvarez-Croda D. M. Stoica B. A. Faden A. I. Loane D. J. (2016). NOX2 drives M1-like microglial/macrophage activation and neurodegeneration following experimental traumatic brain injury. *Brain Behav. Immun.* 58 291–309. 10.1016/j.bbi.2016.07.158 27477920 PMC5067217

[B55] Lavela S. L. Weaver F. M. Goldstein B. Chen K. Miskevics S. Rajan S. (2006). Diabetes mellitus in individuals with spinal cord injury or disorder. *J. Spinal Cord Med.* 29 387–395. 10.1080/10790268.2006.11753887 17044389 PMC1864854

[B56] Li D. X. Wang C. N. Wang Y. Ye C. L. Jiang L. Zhu X. Y. (2020). NLRP3 inflammasome-dependent pyroptosis and apoptosis in hippocampus neurons mediates depressive-like behavior in diabetic mice. *Behav. Brain Res.* 391 112684. 10.1016/j.bbr.2020.112684 32454054

[B57] Li G. Xu X. Wang D. Wang J. Wang Y. Yu J. (2011). Microglial activation during acute cerebral infarction in the presence of diabetes mellitus. *Neurol. Sci.* 32 1075–1079. 10.1007/s10072-011-0632-2 21607752

[B58] Li M. Y. Zhu X. L. Zhao B. X. Shi L. Wang W. Hu W. (2019). Adrenomedullin alleviates the pyroptosis of Leydig cells by promoting autophagy via the ROS-AMPK-mTOR axis. *Cell Death Dis.* 10:489. 31222000 10.1038/s41419-019-1728-5 PMC6586845

[B59] Li R. Wang B. Wu C. Li D. Wu Y. Ye L. (2021). Acidic fibroblast growth factor attenuates type 2 diabetes-induced demyelination via suppressing oxidative stress damage. *Cell Death Dis.* 12:107. 33479232 10.1038/s41419-021-03407-2 PMC7819983

[B60] Liang Z. Han L. Sun D. Chen Y. Wu Q. Zhang L. (2019). Chemerin-induced macrophages pyroptosis in fetal brain tissue leads to cognitive disorder in offspring of diabetic dams. *J. Neuroinflammation* 16:226. 31733653 10.1186/s12974-019-1573-6 PMC6858779

[B61] Liao Y. H. Zhang G. H. Jia D. Wang P. Qian N. S. He F. (2011). Spinal astrocytic activation contributes to mechanical allodynia in a mouse model of type 2 diabetes. *Brain Res.* 1368 324–335. 10.1016/j.brainres.2010.10.044 20971097

[B62] Licastro F. Pedrini S. Caputo L. Annoni G. Davis L. J. Ferri C. (2000). Increased plasma levels of interleukin-1, interleukin-6 and alpha-1-antichymotrypsin in patients with Alzheimer’s disease: peripheral inflammation or signals from the brain? *J. Neuroimmunol.* 103 97–102. 10.1016/s0165-5728(99)00226-x 10674995

[B63] Lien W. C. Kuan T. S. Lin Y. C. Liang F. W. Hsieh P. C. Li C. Y. (2016). Patients with neurogenic lower urinary tract dysfunction following spinal cord injury are at increased risk of developing type 2 diabetes mellitus: a population-based cohort study. *Medicine (Baltimore)* 95:e2518. 26765476 10.1097/MD.0000000000002518 PMC4718302

[B64] Liston A. Masters S. L. (2017). Homeostasis-altering molecular processes as mechanisms of inflammasome activation. *Nat. Rev. Immunol.* 17 208–214. 10.1038/nri.2016.151 28163301

[B65] Liu W. Chen Y. Meng J. Wu M. Bi F. Chang C. (2018). Ablation of caspase-1 protects against TBI-induced pyroptosis in vitro and in vivo. *J. Neuroinflammation* 15 48. 29458437 10.1186/s12974-018-1083-y PMC5817788

[B66] Liu X. Zhang Z. Ruan J. Pan Y. Magupalli V. G. Wu H. (2016). Inflammasome-activated gasdermin D causes pyroptosis by forming membrane pores. *Nature* 535 153–158. 10.1038/nature18629 27383986 PMC5539988

[B67] Liu Z. Dai X. Zhang H. Shi R. Hui Y. Jin X. (2020). Gut microbiota mediates intermittent-fasting alleviation of diabetes-induced cognitive impairment. *Nat. Commun.* 11:855. 32071312 10.1038/s41467-020-14676-4 PMC7029019

[B68] Lu F. Lan Z. Xin Z. He C. Guo Z. Xia X. (2020). Emerging insights into molecular mechanisms underlying pyroptosis and functions of inflammasomes in diseases. *J. Cell Physiol.* 235 3207–3221. 10.1002/jcp.29268 31621910 PMC13482838

[B69] Lutty G. A. (2013). Effects of diabetes on the eye. *Invest. Ophthalmol. Vis. Sci.* 54 Orsf81–Orsf87. 24335073 10.1167/iovs.13-12979 PMC3864380

[B70] Mamun A. A. Wu Y. Nasrin F. Akter A. Taniya M. A. Munir F. (2021). Role of pyroptosis in diabetes and its therapeutic implications. *J. Inflamm. Res.* 14 2187–2206. 10.2147/jir.s291453 34079327 PMC8164340

[B71] Meng X. F. Wang X. L. Tian X. J. Yang Z. H. Chu G. P. Zhang J. (2014). Nod-like receptor protein 1 inflammasome mediates neuron injury under high glucose. *Mol. Neurobiol.* 49 673–684. 10.1007/s12035-013-8551-2 24014157

[B72] Metea M. R. Newman E. A. (2007). Signalling within the neurovascular unit in the mammalian retina. *Exp. Physiol.* 92 635–640. 10.1113/expphysiol.2006.036376 17434916 PMC2279186

[B73] Monif M. Burnstock G. Williams D. A. (2010). Microglia: proliferation and activation driven by the P2X7 receptor. *Int. J. Biochem. Cell Biol.* 42 1753–1756. 10.1016/j.biocel.2010.06.021 20599520

[B74] Moran E. P. Wang Z. Chen J. Sapieha P. Smith L. E. Ma J. X. (2016). Neurovascular cross talk in diabetic retinopathy: pathophysiological roles and therapeutic implications. *Am. J. Physiol. Heart Circ. Physiol.* 311 H738–H749. 27473938 10.1152/ajpheart.00005.2016 PMC5142179

[B75] Mrakic-Sposta S. Vezzoli A. Maderna L. Gregorini F. Montorsi M. Moretti S. (2018). R(+)-thioctic acid effects on oxidative stress and peripheral neuropathy in type II diabetic patients: preliminary results by electron paramagnetic resonance and electroneurography. *Oxid. Med. Cell. Longev.* 2018:1767265. 29849866 10.1155/2018/1767265 PMC5914101

[B76] Nakahira K. Haspel J. A. Rathinam V. A. Lee S. J. Dolinay T. Lam H. C. (2011). Autophagy proteins regulate innate immune responses by inhibiting the release of mitochondrial DNA mediated by the NALP3 inflammasome. *Nat. Immunol.* 12 222–230. 10.1038/ni.1980 21151103 PMC3079381

[B77] Nayak D. Roth T. L. Mcgavern D. B. (2014). Microglia development and function. *Annu. Rev. Immunol.* 32 367–402. 24471431 10.1146/annurev-immunol-032713-120240 PMC5001846

[B78] Puyang Z. Feng L. Chen H. Liang P. Troy J. B. Liu X. (2016). Retinal ganglion cell loss is delayed following optic nerve crush in NLRP3 knockout mice. *Sci. Rep.* 6:20998. 26893104 10.1038/srep20998 PMC4759563

[B79] Qiu Z. He Y. Ming H. Lei S. Leng Y. Xia Z. Y. (2019). Lipopolysaccharide (LPS) aggravates high glucose- and hypoxia/reoxygenation-induced injury through activating ROS-dependent NLRP3 inflammasome-mediated pyroptosis in H9C2 cardiomyocytes. *J. Diabetes Res.* 2019:8151836. 30911553 10.1155/2019/8151836 PMC6398034

[B80] Rahman M. H. Bhusal A. Kim J. H. Jha M. K. Song G. J. Go Y. (2020). Astrocytic pyruvate dehydrogenase kinase-2 is involved in hypothalamic inflammation in mouse models of diabetes. *Nat. Commun.* 11:5906. 33219201 10.1038/s41467-020-19576-1 PMC7680139

[B81] Ramesh G. Maclean A. G. Philipp M. T. (2013). Cytokines and chemokines at the crossroads of neuroinflammation, neurodegeneration, and neuropathic pain. *Mediators Inflamm.* 2013:480739. 23997430 10.1155/2013/480739 PMC3753746

[B82] Ramos-Rodriguez J. J. Jimenez-Palomares M. Murillo-Carretero M. I. Infante-Garcia C. Berrocoso E. Hernandez-Pacho F. (2015). Central vascular disease and exacerbated pathology in a mixed model of type 2 diabetes and Alzheimer’s disease. *Psychoneuroendocrinology* 62 69–79. 10.1016/j.psyneuen.2015.07.606 26254770

[B83] Ren Z. Liang W. Sheng J. Xun C. Xu T. Cao R. (2019). Gal-3 is a potential biomarker for spinal cord injury and Gal-3 deficiency attenuates neuroinflammation through ROS/TXNIP/NLRP3 signaling pathway. *Biosci. Rep.* 39:BSR20192368. 31763668 10.1042/BSR20192368 PMC6923351

[B84] Rühl S. Broz P. (2015). Caspase-11 activates a canonical NLRP3 inflammasome by promoting K(+) efflux. *Eur. J. Immunol.* 45 2927–2936. 10.1002/eji.201545772 26173909

[B85] Sas K. Szabó E. Vécsei L. (2018). Mitochondria, oxidative stress and the kynurenine system, with a focus on ageing and neuroprotection. *Molecules* 23:191. 10.3390/molecules23010191 29342113 PMC6017505

[B86] Savio L. E. B. De Andrade Mello P. Da Silva C. G. Coutinho-Silva R. (2018). The P2X7 receptor in inflammatory diseases: angel or demon? *Front. Pharmacol.* 9:52. 10.3389/fphar.2018.00052 29467654 PMC5808178

[B87] Sborgi L. Rühl S. Mulvihill E. Pipercevic J. Heilig R. Stahlberg H. (2016). GSDMD membrane pore formation constitutes the mechanism of pyroptotic cell death. *EMBO J.* 35 1766–1778. 10.15252/embj.201694696 27418190 PMC5010048

[B88] Schroder K. Zhou R. Tschopp J. (2010). The NLRP3 inflammasome: a sensor for metabolic danger? *Science* 327 296–300. 10.1126/science.1184003 20075245

[B89] Shi C. Wang Q. Rao Z. Shi Y. Wei S. Wang H. (2020). Diabetes induces hepatocyte pyroptosis by promoting oxidative stress-mediated NLRP3 inflammasome activation during liver ischaemia and reperfusion injury. *Ann. Transl. Med.* 8:739. 10.21037/atm-20-1839 32647664 PMC7333130

[B90] Shi J. Gao W. Shao F. (2017). Pyroptosis: gasdermin-mediated programmed necrotic cell death. *Trends Biochem. Sci.* 42 245–254. 10.1016/j.tibs.2016.10.004 27932073

[B91] Shrivastava S. K. Dalko E. Delcroix-Genete D. Herbert F. Cazenave P. A. Pied S. (2017). Uptake of parasite-derived vesicles by astrocytes and microglial phagocytosis of infected erythrocytes may drive neuroinflammation in cerebral malaria. *Glia* 65 75–92. 10.1002/glia.23075 27696532

[B92] Silveira T. N. Gomes M. T. Oliveira L. S. Campos P. C. Machado G. G. Oliveira S. C. (2017). NLRP12 negatively regulates proinflammatory cytokine production and host defense against *Brucella abortus*. *Eur. J. Immunol.* 47 51–59. 10.1002/eji.201646502 27800616 PMC5233573

[B93] Simon D. W. Mcgeachy M. J. Bayır H. Clark R. S. Loane D. J. Kochanek P. M. (2017). The far-reaching scope of neuroinflammation after traumatic brain injury. *Nat. Rev. Neurol.* 13 171–191. 10.1038/nrneurol.2017.13 28186177 PMC5675525

[B94] Singh A. Tetreault L. Kalsi-Ryan S. Nouri A. Fehlings M. G. (2014). Global prevalence and incidence of traumatic spinal cord injury. *Clin. Epidemiol.* 6 309–331. 10.2147/clep.s68889 25278785 PMC4179833

[B95] Song L. Pei L. Yao S. Wu Y. Shang Y. (2017). NLRP3 inflammasome in neurological diseases, from functions to therapies. *Front. Cell. Neurosci.* 11:63. 10.3389/fncel.2017.00063 28337127 PMC5343070

[B96] Strowig T. Henao-Mejia J. Elinav E. Flavell R. (2012). Inflammasomes in health and disease. *Nature* 481 278–286. 10.1038/nature10759 22258606

[B97] Sun J. Wang F. Ling Z. Yu X. Chen W. Li H. (2016). *Clostridium butyricum* attenuates cerebral ischemia/reperfusion injury in diabetic mice via modulation of gut microbiota. *Brain Res.* 1642 180–188. 10.1016/j.brainres.2016.03.042 27037183

[B98] Tseng H. H. Vong C. T. Kwan Y. W. Lee S. M. Hoi M. P. (2016). TRPM2 regulates TXNIP-mediated NLRP3 inflammasome activation via interaction with p47 phox under high glucose in human monocytic cells. *Sci. Rep.* 6:35016. 27731349 10.1038/srep35016 PMC5059733

[B99] Tu Y. Guo C. Song F. Huo Y. Geng Y. Guo M. (2019). Mild hypothermia alleviates diabetes aggravated cerebral ischemic injury via activating autophagy and inhibiting pyroptosis. *Brain Res. Bull.* 150 1–12. 10.1016/j.brainresbull.2019.05.003 31082455

[B100] Vande Walle L. Lamkanfi M. (2016). Pyroptosis. *Curr. Biol.* 26 R568–R572. 27404251 10.1016/j.cub.2016.02.019

[B101] Viganò E. Diamond C. E. Spreafico R. Balachander A. Sobota R. M. Mortellaro A. (2015). Human caspase-4 and caspase-5 regulate the one-step non-canonical inflammasome activation in monocytes. *Nat. Commun.* 6:8761. 26508369 10.1038/ncomms9761 PMC4640152

[B102] Voukali E. Shotton H. R. Lincoln J. (2011). Selective responses of myenteric neurons to oxidative stress and diabetic stimuli. *Neurogastroenterol. Motil.* 23 964–e411. 21914042 10.1111/j.1365-2982.2011.01778.x

[B103] Walsh J. G. Muruve D. A. Power C. (2014). Inflammasomes in the CNS. *Nat. Rev. Neurosci.* 15 84–97. 10.1038/nrn3638 24399084

[B104] Wang B. N. Wu C. B. Chen Z. M. Zheng P. P. Liu Y. Q. Xiong J. (2021). DL-3-n-butylphthalide ameliorates diabetes-associated cognitive decline by enhancing PI3K/Akt signaling and suppressing oxidative stress. *Acta Pharmacol. Sin.* 42 347–360. 10.1038/s41401-020-00583-3 33462377 PMC8027654

[B105] Wang C. Zhu L. Yuan W. Sun L. Xia Z. Zhang Z. (2020). Diabetes aggravates myocardial ischaemia reperfusion injury via activating Nox2-related programmed cell death in an AMPK-dependent manner. *J. Cell Mol. Med.* 24 6670–6679. 32351005 10.1111/jcmm.15318 PMC7299688

[B106] Wang F. Xu C. Reece E. A. Li X. Wu Y. Harman C. (2017). Protein kinase C-alpha suppresses autophagy and induces neural tube defects via miR-129-2 in diabetic pregnancy. *Nat. Commun* 8:15182. 28474670 10.1038/ncomms15182 PMC5424165

[B107] Wang H. Zhou X. Li H. Qian X. Wang Y. Ma L. (2017). Transient receptor potential melastatin 2 negatively regulates LPS-ATP-induced caspase-1-dependent pyroptosis of bone marrow-derived macrophage by modulating ROS production. *Biomed. Res. Int.* 2017:2975648. 29250536 10.1155/2017/2975648 PMC5698788

[B108] Wang L. Q. Zheng Y. Y. Zhou H. J. Zhang X. X. Wu P. Zhu S. M. (2021). LncRNA-Fendrr protects against the ubiquitination and degradation of NLRC4 protein through HERC2 to regulate the pyroptosis of microglia. *Mol. Med.* 27:39. 33858325 10.1186/s10020-021-00299-y PMC8048261

[B109] Wu Y. Q. Xiong J. He Z. L. Yuan Y. Wang B. N. Xu J. Y. (2021). Metformin promotes microglial cells to facilitate myelin debris clearance and accelerate nerve repairment after spinal cord injury. *Acta Pharmacol. Sin.* 10.1038/s41401-021-00759-5 [Epub ahead of print]. 34480113 PMC9160053

[B110] Wuensch T. Thilo F. Krueger K. Scholze A. Ristow M. Tepel M. (2010). High glucose-induced oxidative stress increases transient receptor potential channel expression in human monocytes. *Diabetes* 59 844–849. 10.2337/db09-1100 20068131 PMC2844832

[B111] Xian W. Wu Y. Xiong W. Li L. Li T. Pan S. (2016). The pro-resolving lipid mediator Maresin 1 protects against cerebral ischemia/reperfusion injury by attenuating the pro-inflammatory response. *Biochem. Biophys. Res. Commun.* 472 175–181. 10.1016/j.bbrc.2016.02.090 26915798

[B112] Xu K. Y. Wu C. Y. Tong S. Xiong P. Wang S. H. (2018). The selective Nlrp3 inflammasome inhibitor Mcc950 attenuates lung ischemia-reperfusion injury. *Biochem. Biophys. Res. Commun.* 503 3031–3037. 10.1016/j.bbrc.2018.08.089 30146255

[B113] Xu S. Wang J. Zhong J. Shao M. Jiang J. Song J. (2021). CD73 alleviates GSDMD-mediated microglia pyroptosis in spinal cord injury through PI3K/AKT/Foxo1 signaling. *Clin. Transl. Med.* 11:e269. 33463071 10.1002/ctm2.269 PMC7774461

[B114] Xu S. Zhu W. Shao M. Zhang F. Guo J. Xu H. (2018). Ecto-5’–nucleotidase (CD73) attenuates inflammation after spinal cord injury by promoting macrophages/microglia M2 polarization in mice. *J. Neuroinflammation* 15:155. 29788960 10.1186/s12974-018-1183-8 PMC5964922

[B115] Xue Y. Enosi Tuipulotu D. Tan W. H. Kay C. Man S. M. (2019). Emerging activators and regulators of inflammasomes and pyroptosis. *Trends Immunol.* 40 1035–1052. 10.1016/j.it.2019.09.005 31662274

[B116] Yanagisawa S. Katoh H. Imai T. Nomura S. Watanabe M. (2019). The relationship between inflammasomes and the endoplasmic reticulum stress response in the injured spinal cord. *Neurosci. Lett.* 705 54–59. 10.1016/j.neulet.2019.04.033 31004708

[B117] Yang F. Qin Y. Wang Y. Meng S. Xian H. Che H. (2019). Metformin inhibits the NLRP3 inflammasome via AMPK/mTOR-dependent effects in diabetic cardiomyopathy. *Int. J. Biol. Sci.* 15 1010–1019. 10.7150/ijbs.29680 31182921 PMC6535781

[B118] Yang H. Sloan G. Ye Y. Wang S. Duan B. Tesfaye S. (2019). New perspective in diabetic neuropathy: from the periphery to the brain, a call for early detection, and precision medicine. *Front. Endocrinol. (Lausanne)* 10:929. 10.3389/fendo.2019.00929 32010062 PMC6978915

[B119] Ye L. Li G. Goebel A. Raju A. V. Kong F. Lv Y. (2020). Caspase-11-mediated enteric neuronal pyroptosis underlies Western diet-induced colonic dysmotility. *J. Clin. Invest.* 130 3621–3636. 10.1172/jci130176 32484462 PMC7324173

[B120] Yokoyama S. Cai Y. Murata M. Tomita T. Yoneda M. Xu L. (2018). A novel pathway of LPS uptake through syndecan-1 leading to pyroptotic cell death. *Elife* 7:e37854. 30526845 10.7554/eLife.37854 PMC6286126

[B121] Yu X. Ma X. Lin W. Xu Q. Zhou H. Kuang H. (2021). Long noncoding RNA MIAT regulates primary human retinal pericyte pyroptosis by modulating miR-342-3p targeting of CASP1 in diabetic retinopathy. *Exp. Eye Res.* 202:108300. 10.1016/j.exer.2020.108300 33065089

[B122] Yu Z. W. Zhang J. Li X. Wang Y. Fu Y. H. Gao X. Y. (2020). A new research hot spot: The role of NLRP3 inflammasome activation, a key step in pyroptosis, in diabetes and diabetic complications. *Life Sci.* 240:117138. 10.1016/j.lfs.2019.117138 31809715

[B123] Zendedel A. Johann S. Mehrabi S. Joghataei M. T. Hassanzadeh G. Kipp M. (2016). Activation and regulation of NLRP3 inflammasome by intrathecal application of SDF-1a in a spinal cord injury model. *Mol. Neurobiol.* 53 3063–3075. 10.1007/s12035-015-9203-5 25972240

[B124] Zeng Z. Zheng Q. Chen J. Tan X. Li Q. Ding L. (2020). FGF21 mitigates atherosclerosis via inhibition of NLRP3 inflammasome-mediated vascular endothelial cells pyroptosis. *Exp. Cell Res.* 393:112108. 10.1016/j.yexcr.2020.112108 32445748

[B125] Zhan J. F. Huang H. W. Huang C. Hu L. L. Xu W. W. (2020). Long non-coding RNA NEAT1 regulates pyroptosis in diabetic nephropathy via mediating the miR-34c/NLRP3 axis. *Kidney Blood Press. Res.* 45 589–602. 10.1159/000508372 32721950

[B126] Zhang J. Jiang N. Zhang L. Meng C. Zhao J. Wu J. (2020). NLRP6 expressed in astrocytes aggravates neurons injury after OGD/R through activating the inflammasome and inducing pyroptosis. *Int. Immunopharmacol.* 80:106183. 10.1016/j.intimp.2019.106183 31927506

[B127] Zhang X. Fu Y. Li H. Shen L. Chang Q. Pan L. (2018). H3 relaxin inhibits the collagen synthesis via ROS- and P2X7R-mediated NLRP3 inflammasome activation in cardiac fibroblasts under high glucose. *J Cell Mol. Med.* 22 1816–1825. 10.1111/jcmm.13464 29314607 PMC5824385

[B128] Zheng G. Zhan Y. Wang H. Luo Z. Zheng F. Zhou Y. (2019). Carbon monoxide releasing molecule-3 alleviates neuron death after spinal cord injury via inflammasome regulation. *EBioMedicine* 40 643–654. 10.1016/j.ebiom.2018.12.059 30612943 PMC6412161

[B129] Zhong Z. Umemura A. Sanchez-Lopez E. Liang S. Shalapour S. Wong J. (2016). NF-κB restricts inflammasome activation via elimination of damaged mitochondria. *Cell* 164 896–910. 10.1016/j.cell.2015.12.057 26919428 PMC4769378

[B130] Zhou K. Shi L. Wang Y. Chen S. Zhang J. (2016). Recent advances of the NLRP3 inflammasome in central nervous system disorders. *J. Immunol. Res.* 2016:9238290. 27652274 10.1155/2016/9238290 PMC5019917

[B131] Zhou X. Wang Q. Nie L. Zhang P. Zhao P. Yuan Q. (2020). Metformin ameliorates the NLPP3 inflammasome mediated pyroptosis by inhibiting the expression of NEK7 in diabetic periodontitis. *Arch. Oral. Biol.* 116:104763. 32480011 10.1016/j.archoralbio.2020.104763

[B132] Zychlinsky A. Prevost M. C. Sansonetti P. J. (1992). *Shigella flexneri* induces apoptosis in infected macrophages. *Nature* 358 167–169. 10.1038/358167a0 1614548

